# Effect of the Crystallization Process on the Marginal and Internal Gaps of Lithium Disilicate CAD/CAM Crowns

**DOI:** 10.1155/2016/8635483

**Published:** 2016-03-31

**Authors:** Jae-Hong Kim, Seunghan Oh, Soo-Hyuk Uhm

**Affiliations:** ^1^Department of Dental Technology, School of Medical and Public Health, Kyungdong University, 815 Gyeonhwon-ro, Wonju, Gangwondo 24695, Republic of Korea; ^2^Department of Dental Biomaterials, College of Dentistry, Wonkwang University, 344-2 Shinyong-dong, Iksan, Jeonbuk 570-749, Republic of Korea; ^3^Department and Research Institute of Dental Biomaterials and Bioengineering, Yonsei University College of Dentistry, 50-1 Yonsei-ro, Seodaemun-gu, Seoul 120-752, Republic of Korea; ^4^DNV GL Business Assurance Korea Ltd., 18th Floor, Kyobo Building, 1 Jong-ro, Jongno-gu, Seoul 03154, Republic of Korea

## Abstract

The aim of this study is to quantify the effect of the crystallization process on lithium disilicate ceramic crowns fabricated using a computer-aided design/computer-aided manufacturing (CAD/CAM) system and to determine whether the effect of crystallization is clinically acceptable by comparing values of fit before and after the crystallization process. The mandibular right first molar was selected as the abutment for the experiments. Fifteen working models were prepared. Lithium disilicate crowns appropriate for each abutment were prepared using a commercial CAD/CAM system. Gaps in the marginal area and 4 internal areas of each crown were measured twice—before and after crystallization—using the silicone replica technique. The mean values of fit before and after crystallization were analyzed using a paired *t*-test to examine whether the conversion that occurred during crystallization affected marginal and internal gaps (*α* = 0.05). Gaps increased in the marginal area and decreased in the internal areas after crystallization. There were statistically significant differences in all of the investigated areas (*P* < 0.05). None of the values for marginal and internal fit of lithium disilicate CAD/CAM crowns after crystallization exceeded 120 *μ*m, which is the clinically acceptable threshold.

## 1. Introduction

Prosthodontic treatment requires appropriate functionality, precision, and esthetics. Porcelain-fused-to-metal (PFM) crowns and all-ceramic restorations have been widely used in prosthodontics, especially for esthetic purposes. In these treatment methods, the precision of the prosthesis depends on the proficiency of the dental technician. Errors may occur during the fabrication process and can reduce the accuracy of the prosthesis and affect its marginal and internal fit [1, 2]. Thus, there has been a growing need to solve the problems associated with manual fabrication of prostheses and to produce consistent and high-quality prostheses. As a result, automated computer-aided design/computer-aided manufacturing (CAD/CAM) technology has been applied to dentistry.

Advances in digital technology and the introduction of dental CAD/CAM systems have provided an opportunity to change the traditional methods of taking dental impressions and manually fabricating prostheses. In the past, the production of esthetic restorations required at least 3 visits to a clinic. However, the current use of clinic-based intraoral digital impression systems and computer-based design and production processes for prostheses has made it possible to place esthetic indirect restorations in a single visit by completing all dental laboratory procedures within a short time. The time taken to produce simple esthetic restorations has been reduced to approximately 1 hour [3].

The development of dental CAD/CAM systems has enabled manufacturing of accurate prostheses. Accurate and detailed processing is required to generate precise prostheses. Each part of the process—from tooth preparation to cementation of the prosthesis—can affect the fit. In general, errors occur during the designing and milling processes conducted within the dental CAD/CAM system software based on the STL (stereolithography) extension files and during sintering and shrinkage of the ceramic material [4]. Based on information from the dental model that has been entered, processes, including blocking out of undercuts and manual entry of incomplete margins and air bubbles, are conducted within the design software, and errors can arise during these steps [5]. During the milling process, defects and wear of the bur and the breaking-off of diamond particles from the diamond bur cause errors; not only does this create rough ceramic surfaces but it also leads to cracks in sharp sections of the margins, thus increasing marginal gaps. Moreover, errors can also arise if the bur fractures during the milling process, because of vibration of the milling machine and shaking along the axis of rotation [6, 7].

Lithium disilicate ceramics undergo a crystallization process during the generation of all-ceramic crowns. Based on the results reported by Wiedhahn [8], the effects of the crystallization process can be verified by checking the marginal, proximal, and occlusal fit after the milling procedures. Shrinkage of approximately 0.2–0.3% encountered during the crystallization process does not affect the fit of single crowns. Further, another study reported that the postsintering shrinkage of glass-ceramic composite material does not greatly affect the maintenance of “good fit,” because the material is extremely fine [9]. Lithium disilicate ceramics are available for single crowns of anterior as well as posterior teeth and have been used for various purposes such as single crowns for implants, inlays, onlays, and laminate veneer prostheses, because they have the advantage of minimal linear shrinkage [10].

On the other hand, it has been reported that the high temperature necessary for the crystallization process causes problems with the dimensional stability of lithium disilicate in dental prostheses. Glass-ceramic prostheses undergo crystallization and unexpected manufacturing errors may be caused by microstructural inhomogeneity during this process [11]. No practical study has investigated and verified the marginal and internal fit of prostheses before and after the crystallization process, although previous reports have discussed these factors.

The objective of this study was to analyze the effect of the crystallization process on lithium disilicate ceramic crowns fabricated using the Chairside Economical Restoration of Esthetic Ceramics or CEramic REConstuction (CEREC) CAD/CAM system. This was accomplished by comparing the marginal and internal fit values of partially crystallized crowns with the corresponding values obtained after completion of the crystallization process. A further objective was to reconfirm the clinical acceptability of crystallized lithium disilicate crowns.

## 2. Materials and Methods

**Figure 1:**
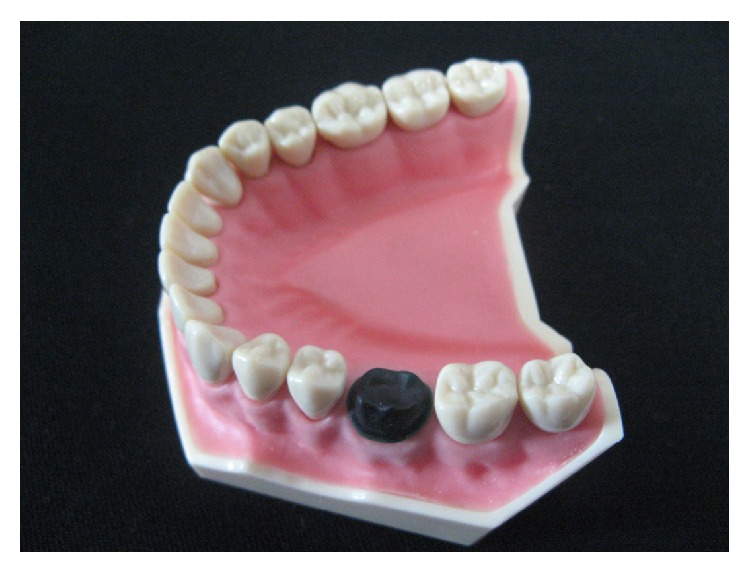
Master model.

For this study, a dentulous mandibular cast (Frasaco AG-3 GmbH, Tettnang, Germany) was selected. After digitizing the cast using a commercially available noncontact white light scanner (Identica, Medit Co. Ltd., Seoul, Korea), an abutment was designed and digitally prepared on the right mandibular first molar using CAD/CAM software (Sensable, Wilmington, NC, USA). In accordance with the manufacturer's instructions, the cervical margin was prepared with a chamfer depth of 1.2 mm. The axial surfaces were cut to provide a 6° taper. According to the information from the digital model and the abutment design, the master cast was fabricated by printing light-cured resin using a three-dimensional (3D) printing device (ProJet*™* DP 3000, 3D Systems, Rock Hill, SC, USA) (Figure 1).

Fifteen full-arch impressions of the master cast were made using light and heavy body polyvinylsiloxane impression materials (Aquasil Ultra XLV, Dentsply International Inc., Milford, DE, USA) in stock metal trays. The same number of replica models was fabricated using high-strength dental stone (Fujirock EP, GC Corporation, Tokyo, Japan) according to the manufacturer's instructions.

After making optical impressions of the abutments and their adjacent teeth using an intraoral scanner (CEREC Bluecam, Sirona Dental Systems GmbH, Bensheim, Germany), the restorations were designed and adjusted using the CEREC 3D system (Sirona Dental Systems GmbH, Bensheim, Germany). Glass-ceramic blocks of lithium disilicate (IPS e.max CAD HT, Ivoclar Vivadent AG, Schaan, Liechtenstein) were milled using the CEREC MC XL system (Sirona Dental Systems GmbH, Bensheim, Germany) after completion of the design of the all-ceramic crowns.

To minimize the excessive wear of diamond burs upon material impact, the milling procedure was performed in the partially crystallized state. Crystallization was performed in a porcelain furnace (P300, Ivoclar Vivadent, Schaan, Liechtenstein) at 850°C for approximately 30 minutes, in accordance with the manufacturer's instructions. IPS Object Fix Putty (Ivoclar Vivadent, Schaan, Liechtenstein) was used along with exclusive trays and pins.

**Figure 2:**
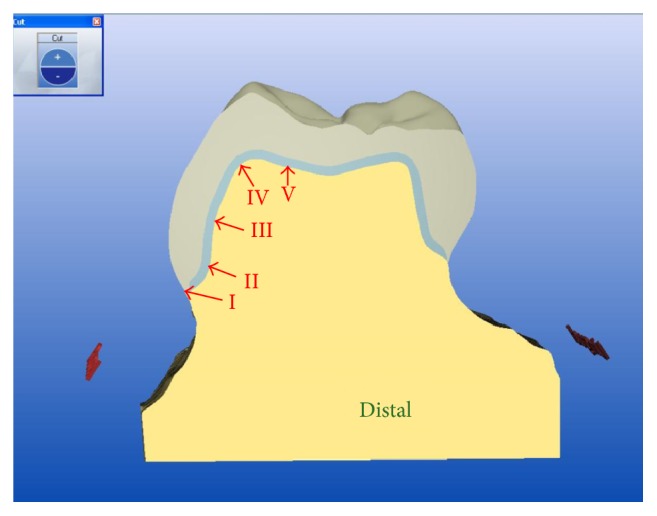
Schematic representation of the measurement areas (indicated by Roman numerals) in a cross section of a lithium disilicate CAD/CAM crown. I: marginal area; II: chamfer area; III: axial area; IV: axioocclusal angle; V: occlusal area; CAD/CAM: computer-aided design/computer-aided manufacture.

To evaluate the marginal and internal fit of crowns, this study modified the definitions used by Reich et al. [12] and Colpani et al. [13]. The fit of the restorations was measured from 4 directions using labiolingual and mesiodistal sections. Five measurement points were investigated: (1) marginal area (area I), (2) chamfer area (area II), (3) axial area (area III), (4) axioocclusal angle (area IV), and (5) occlusal area (area V) (Figure 2).

The “replica technique” proposed by Molin and Karlsson [14] was used for measurement of fit. This technique measures the thickness of a section of a silicone replica obtained after filling the gap between the prosthesis and the abutment with silicone material. A light body polyvinylsiloxane impression material (Aquasil Ultra XLV, Dentsply DeTrey GmbH, Konstanz, Germany) was used to fill inside the crown. In order to standardize finger pressure, the study model was placed on the center of the scale, and approximately 50 N of finger pressure was applied to the occlusal surface for about 2 minutes and 30 seconds, until the light body silicone completely hardened [15, 16]. In order to deliver a uniform amount of force to all points, the pressure was controlled to prevent the scale plate from leaning toward any one side. Finger pressure was applied on all specimens, to best reflect the actual clinical situations that can occur when dental prostheses are performed in the cementation procedure.

**Figure 3:**
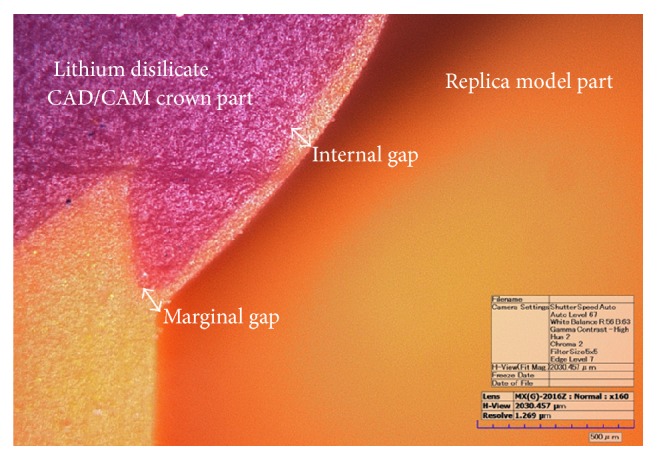
Measurement of marginal and internal gaps using a digital microscope (magnification ×160).

To stabilize its shape, the light body polyvinylsiloxane was supported by a medium body polyvinylsiloxane impression material (Aquasil Ultra Monophase, Dentsply DeTrey GmbH, Konstanz, Germany). Thereafter, the light-medium body complex was additionally covered with a heavy body polyvinylsiloxane impression material (Aquasil Ultra Monophase; Dentsply DeTrey GmbH, Konstanz, Germany) in a separate tray. The tray was prepared in the form of a square box of baseplate wax with a uniform length, width, and height of 20 mm each. To guarantee accurate and uniform cutting of all the specimens, the silicone complex was cut at the exact center of the buccolingual and mesiodistal directions, using a razor blade and a ruler. The thickness of the light body silicone in each section was then measured by a single examiner at a magnification of ×160 using a digital microscope (KH-7700, HIROX, Tokyo, Japan) (Figure 3).

To obtain reliable marginal and internal fit values, each of the 15 specimens was measured at 5 points from 4 directions, yielding 300 measurements in total. In the first set of measurements, the marginal and internal fit between the crown and the abutment were measured in the partially crystallized state (before crystallization). In the second set of measurements, the fit between the crown and the abutment was measured after completion of the crystallization process (after crystallization), thus yielding 600 measurements in all. In accordance with the definition provided by Holmes et al. [17], the vertical distance from the abutment to the prosthesis was measured.

SPSS 12.0 for Windows (SPSS Inc., Chicago, IL, USA) was used for statistical analysis. To evaluate the marginal and internal fit after crystallization and to analyze whether there were significant differences in the mean values between the 2 groups, a parametric paired *t*-test was performed after a Shapiro-Wilk test for normality (*P* = 0.093, before crystallization; *P* = 0.071, after crystallization). Statistical significance was defined at *P* < 0.05.

## 3. Results

**Figure 4:**
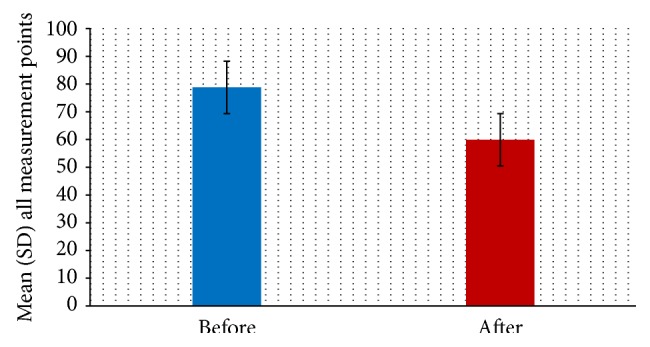
The total mean and standard deviation (SD) values for all measurement points (*n* = 300) of the lithium disilicate CAD/CAM crowns, after crystallization (*P* < 0.05).

The mean and standard deviation values of the fit in the marginal and internal areas before and after the crystallization process indicated that the marginal gap was larger and the internal gap was smaller after the crystallization process and that this difference was statistically significant (*P* < 0.05) (Table 1). The overall mean (average of marginal and internal fit) and standard deviation values for all measurement points before and after crystallization were 78.82 ± 26.63*μ*m and 59.91 ± 30.38*μ*m, respectively, with a statistically significant difference in all of the areas evaluated (*P* < 0.05) (Figure 4).

## 4. Discussion

Several studies have examined the marginal and internal fit of fixed partial dentures prepared using the CEREC system. With regard to the CEREC inLab system (Sirona Dental Systems GmbH, Bensheim, Germany), relatively small marginal gaps of 43 ± 23*μ*m and moderately large internal gaps ranging from 82 ± 49*μ*m to 114 ± 58*μ*m in Empress II crown copings (Ivoclar Vivadent, Schaan, Liechtenstein) have been reported [7]. It has also been reported that the all-ceramic crowns of the IPS e.max CAD system prepared using the CEREC 3D system were clinically acceptable, with 100–200 *μ*m of average marginal fit [18] In an earlier study that measured the cement thickness of 20 lithium disilicate crowns in a patient's oral cavity using a light body silicone, an average cement thickness range of 100–284 *μ*m was reported [12], and the authors insisted that the film thicknesses were close to meeting clinical acceptability. The production of all-ceramic crowns using the CEREC system achieves effective results and reliable clinical prognosis by simplifying the process. In a study evaluating the 2-year prognosis of lithium disilicate crowns prepared using the CEREC AC system, no major problem was reported [19]. Another study showed concordant results in that secondary caries or fractures of single crowns were not observed for 4 years and the success rate was 96.3% [20].

Since 1985, casting and sintering methods have been rapidly replaced by methods that use dental CAD/CAM systems; this trend will continue in the future [21]. Recently, in the field of dentistry, the use of metal-ceramic crowns has decreased, and the use of all-ceramic crowns has shown an increasing trend, due to their improved aesthetics and biocompatibility. Although all-ceramic crowns have their advantages with regard to biocompatibility, aesthetics, chemical resistance, and reduction of plaque accumulation, they have relatively high brittleness and low tensile strength, which previously limited their application, particularly in posterior fixed partial dentures [22, 23]. To compensate for this disadvantage, particulate-reinforced ceramics have been invented, using materials such as aluminum oxide, leucite, lithium disilicate, and zirconium oxide, and these ceramics have been used not only in the restoration of posterior teeth but also in longer fixed partial dentures; however, the fit of the prosthesis is a critical factor in determining the success of a treatment, owing to the nature of the dental prosthesis. The precision of prostheses fabricated using CAD/CAM systems has been the subject of some controversy. Therefore, this study is significant because it analyzed the effects of the crystallization process on the marginal and internal areas during the production of lithium disilicate CAD/CAM crowns, which are one of the more frequently used all-ceramic crowns.

The overall mean and standard deviation values of the marginal and internal fit were 78.82 ± 26.63*μ*m in the partially crystallized state and 59.91 ± 30.38*μ*m in the crystallized state. A paired *t*-test confirmed that these values showed a statistically significant difference in fit (*P* < 0.05). For the occlusal surface area in particular, the fit changed from 122.06 ± 33.97*μ*m (which exceeded the clinically acceptable range) to 88.01 ± 26.34*μ*m (which is within the clinically acceptable range). On the other hand, the fit in the marginal area worsened from 91.63 ± 34.53*μ*m to 103.12 ± 25.46*μ*m. None of the values measured exceeded after crystallization 120 *μ*m, which is the clinically acceptable threshold for fit in a fixed dental prosthesis. Although there is no clear consensus on the acceptable standard or threshold value for a clinically acceptable fit in a fixed dental prosthesis [17, 24, 25], the limit proposed by most researchers is 120 *μ*m, as indicated by McLean and von Fraunhofer [25]. After observing 1000 prostheses for 5 years, they reported that 120 *μ*m is an appropriate limit for clinical acceptability. However, another study reported that most clinicians preferred a fit of 50 *μ*m or less and regarded 100 *μ*m as a clinically acceptable limit that might provide durability in the oral cavity [17, 24].

Previous studies have suggested that the sintering process used in the manufacture of all-ceramic crowns has an effect on marginal fit. Piddock and Qualtrough [26] reported that shrinkage caused problems with marginal fit in ceramic restorations, and the results of the present study corroborate their findings. Furthermore, Kunii et al. [6] reported on the effects of final sintering during the manufacturing process of partially crystallized zirconia prostheses and confirmed that the cement layer of the zirconia core was larger after sintering than the set value. They concluded that expansion of this cement layer was caused by anisotropic shrinkage and confirmed that sintering shrinkage occurred to a lesser extent along the long axis of the tooth than along the horizontal axis. These properties cause dimensional changes in zirconia prostheses due to anisotropic shrinkage even when zirconia blocks with homogeneous properties go through the sintering process.

During the crystallization process, the prosthesis is milled through the dental CAD/CAM system and is fired at 840°C for 25 minutes. At the time of milling, the material is partially crystallized (lithium metasilicate), and the size of particles generally ranges between 0.2 *μ*m and 1 *μ*m, with a flexural strength of 130 MPa. After the crystallization phase, the size of the particles increases under control, from 0.5 *μ*m to 5 *μ*m. Through such modification processes, prismatic glass ceramics are formed and dispersed over the glassy matrix. Due to this change, the flexural strength of the restoration increases 1.7-fold to 360 MPa [27]. In addition, a 0.2% linear shrinkage occurs during this process, as the crystal spacing becomes denser while the proportion of fine lithium disilicate crystals within the glassy matrix, which previously was approximately 40%, increases to 70% after complete crystallization. Such changes can affect the overall fit of the dental prosthesis, increasing marginal gaps while decreasing internal gaps. Moreover, the block, which appears blue when partially crystallized, acquires the characteristic color of dental prostheses [10].

In the present study, none of the experimental values exceeded the clinically acceptable threshold of 120 *μ*m proposed by McLean and von Fraunhofer [25]. Based on these results, this study could not confirm that the 0.2% shrinkage generated by the crystallization process affected the fit of the final prostheses. Further, it is not possible to determine accurately the specific direction of distortion. Studies using glass-ceramic composite materials have been reported frequently in the field of material engineering [9]. However, insufficient research has been conducted on the possible changes in fit following the crystallization processes that are involved in the production of dental prostheses. The results of this experiment are limited to single crowns. Future studies examining the effects of the crystallization process on fit in more complex shapes and actual clinical models will help to further our understanding of this subject.

## 5. Conclusions

Within the limitations of this study, during the production of lithium disilicate CAD/CAM crowns, differences in the marginal and internal fit measured before and after the crystallization process were confirmed to be statistically significant in all areas (*P* < 0.05). Most of the values that were obtained after the crystallization process were within the clinically acceptable range suggested by many researchers. Therefore, it cannot be concluded that crystallization has a major effect on fit.

## Figures and Tables

**Table 1:** Mean and standard deviation values of marginal and internal gaps in computer-aided design/computer-aided manufacturing (CAD/CAM) lithium disilicate crowns (*n* = 15) before and after the crystallization process at each of the 5 points investigated (measured in micrometers [*µ*m]).

Measurement point	Landmark^a^	Before crystallization	After crystallization	*P* value
I	MA	91.63 (34.53)	103.12 (25.46)	0.016
II	CA	75.77 (11.94)	47.86 (9.84)	0.001
III	AA	45.23 (9.17)	24.74 (7.82)	0.001
IV	AOA	59.45 (13.06)	35.86 (11.86)	0.001
V	OA	122.06 (33.97)	88.01 (26.34)	0.001

^a^MA: marginal area; CA: chamfer area; AA: axial area; AOA: axioocclusal angle; OA: occlusal surface area.
